# Experimental feline enteric coronavirus infection reveals an aberrant infection pattern and shedding of mutants with impaired infectivity in enterocyte cultures

**DOI:** 10.1038/srep20022

**Published:** 2016-01-29

**Authors:** Lowiese M. B. Desmarets, Ben L. Vermeulen, Sebastiaan Theuns, Nádia Conceição-Neto, Mark Zeller, Inge D. M. Roukaerts, Delphine D. Acar, Dominique A. J. Olyslaegers, Marc Van Ranst, Jelle Matthijnssens, Hans J. Nauwynck

**Affiliations:** 1 Department of Virology, Parasitology and Immunology, Faculty of Veterinary Medicine, Ghent University, Salisburylaan 133, B-9820 Merelbeke, Belgium; 2 Laboratory of Clinical Virology, Department of Microbiology and Immunology, Rega Institute for Medical Research, Minderbroedersstraat 10, B-3000 Leuven, Belgium; 3 Laboratory of Viral Metagenomics, Department of Microbiology and Immunology, Rega Institute for Medical Research, Minderbroedersstraat 10, B-3000 Leuven, Belgium

## Abstract

Feline infectious peritonitis (FIP) results from mutations in the viral genome during a common feline enteric coronavirus (FECV) infection. Since many virological and immunological data on FECV infections are lacking, the present study investigated these missing links during experimental infection of three SPF cats with FECV strain UCD. Two cats showed mild clinical signs, faecal shedding of infectious virus from 4 dpi, a cell-associated viraemia at inconsistent time points from 5 dpi, a highly neutralising antibody response from 9 dpi, and no major abnormalities in leukocyte numbers. Faecal shedding lasted for 28–56 days, but virus shed during this stage was less infectious in enterocyte cultures and affected by mutations. Remarkably, in the other cat neither clinical signs nor acute shedding were seen, but virus was detected in blood cells from 3 dpi, and shedding of non-enterotropic, mutated viruses suddenly occurred from 14 dpi onwards. Neutralising antibodies arose from 21 dpi. Leukocyte numbers were not different compared to the other cats, except for the CD8^+^ regulatory T cells. These data indicate that FECV can infect immune cells even in the absence of intestinal replication and raise the hypothesis that the gradual adaptation to these cells can allow non-enterotropic mutants to arise.

Feline coronaviruses (FCoVs) occur as two pathotypes, associated with either enteric or systemic diseases in cats. Feline enteric coronavirus (FECV) is an enterotropic virus, ubiquitously present in the cat population^1^,^2^. The enteritis caused by the intestinal replication can manifest as a transient anorexia, weight loss and/or diarrhoea, but clinical signs are often too mild to be noticed^1^,^3^,^4^. Feline infectious peritonitis virus (FIPV) most likely arises from FECV by accumulation of mutations in individually infected cats^5^,^6^,^7^,^8^,^9^,^10^,^11^. These yet not fully characterized mutations abrogate the enterocyte tropism but provide the virus with tools to productively replicate in monocytes/macrophages, causing a highly fatal systemic disease, feline infectious peritonitis (FIP), which is characterised by a diffuse vasculitis, polyserositis and severe lymphopaenia^12^,^13^,^14^,^15^,^16^,^17^. To date, it remains unknown where, when, and how this pathotype switch is induced in FECV-infected cats.

Due to its pathogenic behaviour, FIPV has received considerable attention, and clinical, virological, and immunological parameters during both natural and experimental FIPV infections have frequently been studied^14^,^15^,^16^. The last decade, comprehensive studies on the FIPV parent virus, FECV, have extensively contributed to our current understanding of the epizootiology and pathogenesis^4^,^18^,^19^,^20^, but many crucial virological and immunological data on the FECV-cat interactions are missing to fully understand the behaviour of this FIPV parent virus. Due to the lack of an FECV-susceptible cell line, there is so far no information on the infectivity (and its correlation with RT-qPCR results) of faeces, and on the generation of neutralising antibodies during FECV infections. Feline enterocyte cultures sustaining the replication of FECVs have previously been developed^21^, finally allowing the quantification of enterotropic viruses and neutralising antibodies in *in vivo* experiments. In addition, whereas immune responses during FIP development have been extensively studied^13^,^16^,^17^,^22^, hardly any information is available on the dynamics of several leukocyte subsets during FECV infections. Moreover, although mutations play a key role in the FCoV pathogenesis, too little is known about the viral genome evolution during FECV infections and the impact of these mutations on the infectivity of the faecally shed viruses. Therefore, this study aimed at further broadening our knowledge on the FECV pathogenesis, by monitoring various clinical, virological (genome evolution, virus infectivity in enterocyte cultures, and onset and duration of viraemia), and immunological (presence of neutralising antibodies and the dynamics of several leukocyte subsets) parameters in the 3 months following inoculation of three specific pathogen free (SPF) cats with FECV strain UCD.

## Results

### Clinical signs

**Figure 1:**
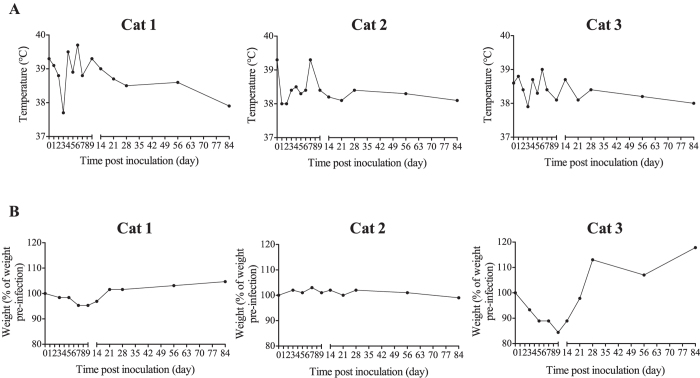
Clinical parameters followed during the entire FECV UCD infection course. (**A**) Rectal temperature was monitored daily during the first week, and on day 9, 14, 21, 28, 56, and 84 pi. (**B**) Body weight was measured at day 0, 3, 5, 7, 9, 14, 21, 28, 56, and 84, and expressed relative to the weight before inoculation.

Mild clinical signs were seen in cat 1 and cat 3 during the first week after inoculation. They consisted of diminished appetite and moderate weight loss, to 95.4 and 88.4% of the initial weight for cat 1 and 3, respectively. Cat 1 also showed an increased body temperature at 4 (39.5 °C) and 6 (39.7 °C) dpi. No diarrhoea or changes in faecal consistency were observed. From day 9, both cats started to recover and reached their original (or slightly higher) weight at 21 dpi. Cat 2 showed no loss of appetite, weight loss or abnormal stool consistency during the entire experiment, but a slightly raised temperature (39.3 °C) was found at 7 dpi (Fig. 1).

### Viral shedding

**Figure 2:**
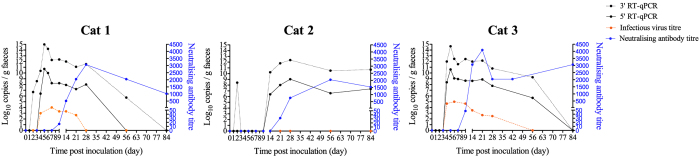
Faecal shedding and neutralising serum antibody response during FECV UCD infection. Faeces (or faecal swabs if faeces were not available) were taken at regular time points pi, and the total amount of viral RNA was quantified by RT-qPCR using either primers targeting the 3′ part of the genome and subgenomic mRNAs (3′ RT-qPCR, black dashed line) or primers against the ORF1b to detect only genomic RNA (5′ RT-qPCR, black line). The amount of infectious virus was determined by titration of faecal suspensions in feline enterocyte cultures (orange dashed line). Neutralising antibody titres were assessed in the serum on day 0, 3, 5, 7, 9, 14, 21, 28, 56, and 84 pi by virus neutralisation assay in enterocytes using FECV UCD (blue line).

Faecal shedding was quantified by 2 different RT-qPCRs and by virus titration in feline enterocyte cultures. These 2 RT-qPCRs were used to assess the overestimation of genomic RNA by the generally used 3′ RT-qPCR^23^, as this RT-qPCR detects not only genomic RNA, but also all subgenomic mRNAs. As shown in Fig. 2, two different excretion patterns were detected. Cat 1 and cat 3 started shedding viral RNA from day 2–4 onwards. For these 2 cats, faecal shedding peaked at 5 dpi, whereupon shedding slightly decreased but remained at high levels until 28 dpi, and even a second small peak was seen at 28 and 21 dpi for cat 1 and 3, respectively. Thereafter, viral RNA levels dropped and both cats had ceased shedding by 84 dpi. Viral RNA quantities were 3–4.3 log_10_ higher with the 3′ RT-qPCR compared to the 5′ RT-qPCR, indicating that only 1/1000 to 1/20000 of all copies detected with the 3′ RT-qPCR are viral genomic RNA copies. Infectivity titration of faecal suspensions in feline enterocyte cultures showed that infectious virus was detectable from day 4 until day 21 (cat 1) or day 28 (cat 3), but that infectious virus titres declined more rapidly than the RT-qPCR titres. In contrast to cat 1 and 3, an aberrant shedding pattern was found in cat 2. At day 2, viral RNA was detected with the 3′ RT-qPCR, but not with the 5′ RT-qPCR. Thereafter, viral RNA was undetectable until 14 dpi. From then, faecal RNA shedding appeared and remained high during the remainder of the experiment, and this cat ultimately stopped shedding 6 months after inoculation (data not shown). Remarkably, no virus replication was observed at any of the time points when faecal samples were inoculated on intestinal epithelial cell cultures.

RNA levels in saliva were the highest in all cats directly after inoculation. Subsequently, the days at which the samples were positive varied considerably between cats, and positive samples always contained very low amounts of RNA (data not shown).

### Immunological parameters

#### Neutralising antibodies

For cat 1 and 3, neutralising antibodies were detected from 9 dpi and peaked at 21 (cat 3) or 28 (cat 1) dpi. In cat 2 with the delayed shedding pattern, similar signs of seroconversion occurred only after the onset of intestinal replication, with the first detectable antibodies appearing at 21 dpi. In all cats, antibody titres remained high until the end of the experiment (Fig. 2).

#### Dynamics of leukocyte subsets

**Figure 3:**
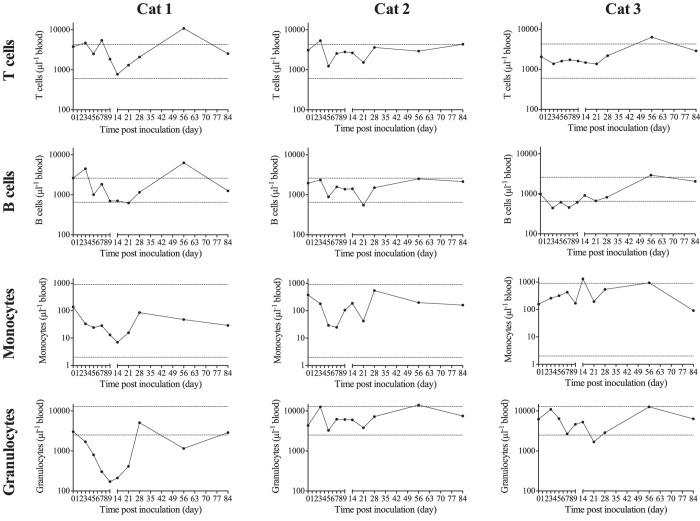
Absolute quantity of different leukocyte subsets during FECV infection. The absolute numbers of T cells, B cells, monocytes, and granulocytes were assessed by flow cytometric analysis of cells present in regularly taken blood samples. Two horizontal dashed lines represent reference values in healthy animals.

For all cats, the absolute number of T cells, B cells, monocytes, and granulocytes were determined in blood taken at regular time points after inoculation. No abnormal leukocyte numbers were noticed in any of the cats, except for a depletion of peripheral granulocytes in cat 1 during the first 3 weeks pi. For each cat, T and B cell numbers followed a similar trend. All cats showed a small decrease in lymphocyte numbers, which started to resolve from 21 dpi, but this recovery phase was much more pronounced in cat 1 and 3 compared to cat 2. Indeed, whereas lymphocyte numbers remained at pre-infection levels for cat 2, both cat 1 and 3 showed a slight lymphocytosis, which coincided with cessation of shedding in both cats. Monocytes of cat 1 and 2 slightly declined to rise back to pre-infection levels at 28 dpi, but numbers always remained within the normal limits (Fig. 3).

**Figure 4:**
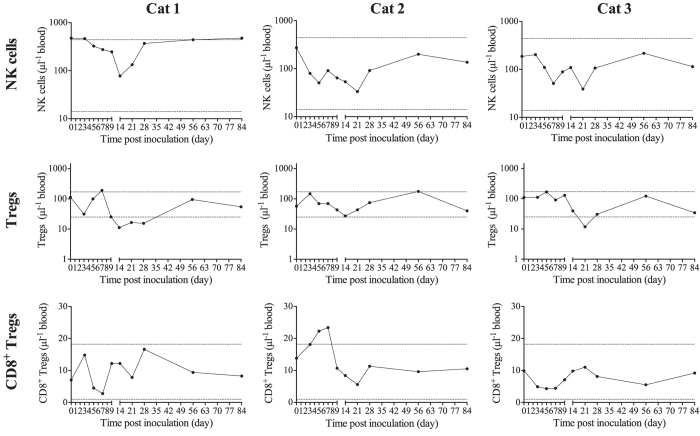
Number of NK cells and Tregs during FECV infection. NK cells, Tregs, and CD8^+^ Tregs were quantified by flow cytometry in regularly taken blood samples. Two horizontal dashed lines represent reference values in healthy animals.

Quantification of natural killer (NK) and regulatory T cells (Tregs) showed no abnormal high or low NK cell- or Treg numbers during the infection. However, some trends were visible. In all cats, NK cells slowly declined until 14 or 21 dpi, whereupon they rose again to pre-infection level at 56 dpi. Treg counts similarly declined and rose in all cats. When analysing a subset of Tregs (CD8^+^ Tregs), which has been associated with suppression of gut immune responses, it was noticed that the delayed shedder had higher numbers of CD8^+^ Tregs, which increased until 7 dpi, whereas the number of CD8^+^ Tregs was slightly decreased during the first week for the other 2 cats (Fig. 4).

### Viraemia

Both cell-free and cell-associated viraemia were assessed at regular time points for all cats, using the 5′ RT-qPCR. No cell-free viraemia was detected, but a cell-associated viraemia was observed at infrequent time points for all cats. In contrast to cat 1 and 3, viraemia in cat 2 was detected before the onset of faecal shedding (3 and 5 dpi), and no longer thereafter (Table 1).

### Analysis of viral genome evolution in faeces

As mentioned above, several inconsistencies between the results for the faecal RNA shedding and the infectivity titration in enterocyte cultures were observed, including a total lack of infectivity for cat 2 during the entire study, and a reduction in *in vitro* enterocyte tropism for cat 1 and 3 from 14–21 dpi onwards. To find an explanation for this discrepancy, the complete genomes of the faecally shed viruses were determined at different time points.

Table 2 shows the nucleotide and amino acid differences between the inoculum and the viruses found at an early shedding stage (day 7, 21, and 9 for cat 1, 2, and 3, respectively). Although inoculated with the same strain, every cat developed its own quasispecies very rapidly. The most striking difference was found in the viruses shed by cat 2, since 83.8% of all reads showed a 101 bp deletion in the 7b gene, resulting in the formation of a truncated 7b protein with only 143 amino acids instead of 206. Surprisingly, this deletion was no longer found at a later time point (84 dpi) (Table 3), suggesting that viruses with an intact 7b protein were in time selected over the mutants containing the deletion. Other amino acid substitutions were found for this cat in nsp2, nsp5, and nsp6. In cat 1, no amino acid substitutions had occurred by 7 dpi, whereas for cat 3, single amino acid substitutions were found in a minority of the viruses in nsp6, nsp9, nsp12, and the spike protein at 9 dpi.

In contrast to the early shedding, viruses shed by all cats at the end of the infection had undergone numerous changes in the viral genome, with the spike protein being the most affected in all cats (Table 3). Most of the mutations in the spike protein occurred in the globular S1 domain. These changes included deletions (cat 1 and 3) and various amino acid substitutions with possibly a high impact on the charge, polarity and/or glycosylation potential. Amino acid 33 was a hotspot for mutation, since the proline residue had changed to serine in cat 1 (thereby adding a potential N-glycosylation site as predicted by the NetNGlyc server) and leucine in cat 2. In cat 3, 90% of all viruses had a deletion of 8 amino acids in this region, whereas the other 10% showed the proline to serine substitution. All other changes in the S1 region of the spike protein differed between the cats, except for the amino acid substitution K665N that occurred in both cat 1 and 2. In contrast to the S1 domain, the S2 domain of the spike was less affected by mutations, as only one amino acid change had occurred in cat 2 and cat 3. One of these mutations (T1107I in cat 2) occurred in the heptad repeat 1 (HR1), a region that was recently shown to be affected by mutations in many FIP cats^24^,^25^. If one or more of these mutations in the spike protein can be linked to the reduced *in vitro* enterocyte tropism requires further investigation. Apart from the spike protein, mutations were also found in other proteins, which again differed between cats. All these results indicate that there is a strong selection pressure during FECV infections, which forces the virus to rapidly evolve, hence resulting in the onset of virus quasispecies that differ among cats.

## Discussion

Feline infectious peritonitis (FIP) arises by mutations in the viral genome during a common feline enteric coronavirus (FECV) infection. However, studies on the latter, parental virus are scarce^1^,^4^,^18^,^19^,^20^, and many crucial data on FECV infections are lacking, which hampers our understanding of the pathogenesis. The present study reports a detailed investigation of various clinical, virological, and immunological parameters during an experimental FECV infection in three cats.

Analysis of the faecal shedding revealed two distinct excretion patterns. In cat 1 and 3, ingestion of the virus resulted in acute shedding of viral RNA, peaking at 5 dpi, followed by a plateau and a small second peak at 21–28 dpi. Finally, shedding declined to undetectable levels at 84 dpi. This RNA excretion pattern is reminiscent of previous reports on experimental FECV infections^4^,^19^,^20^. Virus shedding determined by infectivity titration in feline enterocytes cultures was more transient, since infectivity titres declined already from 14–21 dpi, which made the ratio genomic RNA copies/infectious virus titre increase from 3–4 log_10_ during the first week, to up to 8 log_10_ at 28 dpi. As the decrease in infectivity coincided with the onset of neutralising antibodies in cat 1 and 3, a possible explanation for this lack of correlation is that neutralising antibodies in faeces caused an increased underestimation of infectious virus in the cell culture-based assay. However, it cannot be excluded that this decreased *in vitro* infectivity was caused by one of the mutations found at this stage of the infection. In sharp contrast to these two cats, cat 2 showed a remarkably different and atypical excretion pattern. This cat lacked the acute shedding phase but suddenly started shedding virus from 14 dpi, without ever showing any of the clinical signs associated with intestinal replication (anorexia and weight loss) seen in the other 2 cats or previously reported FECV UCD infected cats^4^. The shedding was also more prolonged compared to the other cats. A delay in faecal shedding has been described in one previous study, reporting no faecal shedding before 10 dpi in a cat inoculated with a weak-positive faecal extract^26^. However, in the present study all cats were infected with a high dose of the same inoculum (10^11.3^ RNA copies), and another previous study reported the successful inoculation of cats with FECV UCD at a dose as low as 10^5.7^ RNA copies, without noticing this delay^4^. Another possible explanation for this delay is that the original inoculation failed, and that this cat became infected later on by inadvertent transmission of the virus shed by one of the other cats. This explanation seems also very unlikely, as 1) cats were housed separately and precautions were taken to avoid inadvertent transmission, 2) FCoV RNA was found in saliva of this cat until 2 days after inoculation (data not shown), 3) viral RNA was found in faeces at day 2, indicating passage of the inoculum without any further infection, and 4) the FECV genome in cat 2 differed markedly from the other cats. Indeed, a surprising finding resulting from full genome sequencing was that 83.8% of all viruses shed by cat 2 showed a 101 bp deletion in the 7b gene, resulting in a clearly shorter translation product (143 amino acids instead of 206). So far, the role of the 7b protein remains enigmatic, but this protein is believed to play a crucial role during natural FCoV infections as it is conserved in field strains^11^,^16^,^27^. Up to now, large 7b deletions (56–406 bp) have only been seen in laboratory strains, whereas the gene is intact or only affected by small deletions (max 12 bp) in field strains (both FECV and FIPV)^28^. Therefore, it is interesting to find that FECV continues to replicate in the absence of an intact 7b, yet there was a positive selection pressure to restore its function as infection progressed. Because no 7b deletion mutants were found in the inoculum, this cannot be the reason why the virus did not replicate in the gut after inoculation. Consequently, the most plausible reason for the lack of acute shedding in cat 2 is that cat-dependent factors (such as harsh digestive conditions, absence of receptors and/or strong innate immunity) restricted the virus to replicate in the intestinal epithelial cells upon oral inoculation, as it is known that some cats are resistant to FECV infection^29^. However, this cat did suddenly start shedding virus from 14 dpi, and these viruses were not only affected by a 7b deletion, they were also no longer infectious in enterocytes, at least *in vitro*, as no additional inoculation studies were done with the faecal suspensions of this cat to confirm this feature. Nevertheless, given the huge differences in infectivity compared to the other 2 cats, it can be questioned if this virus was indeed shed by enterocytes, and not by another cell type residing in the intestinal mucosa, since the gradual adaptation to these non-enterocytes can explain the delay and can result in phenotypic changes induced by mutation or altered post-translational modifications. It is known that FECV is not confined to the intestinal epithelial cells but can also be found at low level throughout the body in cells of the monocytic lineage^19^,^30^. This systemic spread is supported by the cell-associated viraemia detected in the present study, although a recent report indicated that care should be taken when interpreting RT-qPCR-based viraemia data due to the occurrence of false positives at the limits of the assay (from Cq values of 37 onwards)^17^. In cat 2, a cell-associated viraemia with Cq values of 34.2 and 32.7 was noticed at day 3 and 5 pi, respectively, indicating a systemic spread in this cat. This early uptake by (most probably monocytic) immune cells, in addition to the initial lack of replication in enterocytes, can support the idea that the delayed shedding of mutant viruses that completely lacked *in vitro* enterocyte infectivity might have resulted from the gradual adaptation to the replication in locally present, mucosa-associated immune cells. The gradual adaptation to non-enterocytes may also give another explanation for the inconsistencies seen between RT-qPCR titres and infectivity titres at later stages of the infection in the other 2 cats.

Full genome sequencing revealed that the virus was faced with a high selection pressure in all cats, but none of the hitherto described FIPV-specific mutations in the spike and 3c genes were ever found during the entire study^5^,^9^,^31^,^32^. The onset of mutant viruses is not surprising, since RNA viruses generally have a high mutation rate, resulting in the formation of a genetic heterogeneous virus population (quasispecies) that allows these viruses to rapidly evolve by selecting new variants^33^. In the present study, notably the amino-terminal part of the spike protein had extensively mutated in all cats, and two of the three cats even showed small deletions (3 and 8 amino acids in cat 1 and 3, respectively) in this domain. The high mutation rate in the S1 part of the spike protein is not really unexpected, since it is known that this region is an important target for immunological selection, and hence antigenic drift^25^,^27^. However, the presence of deletions and the clearly diminished infectivity of these viruses observed in the present study will make it worthwhile to closer investigate the impact of such mutations. Indeed, to our knowledge, S1 deletions have been observed in various FIPV strains^11^,^31^,^34^, but not in enteric strains, which indicates that this region is potentially required for enterocyte infections, but dispensable for FIP development, as suggested before^34^. Since this region is also a determinant for the enterotropism of the related alphacoronavirus TGEV^35^,^36^, one or more of these mutations may give an explanation for the loss of infectivity in enterocyte cultures. In addition, some of the mutations could have an impact on the glycosylation of the virus, and hence change its interaction with C-type lectins that have been shown to be involved in FIPV infection of monocytes^37^,^38^,^39^. Apart from the amino-terminal spike protein, SNPs were also found in the more distal S1 domain, the S2 domain, nucleocapsid protein, membrane protein, and various non-structural proteins encoded by ORF1a. Although it is difficult at this point to make conclusions on the link between any of the observed mutations and the phenotype changes of the virus, the present study indicates that potentially not all faecally shed viruses in healthy cats are actual enterotropic strains, but rather variant forms with a yet unclear cell tropism. Although no FIPV-associated mutations^5^,^9^,^31^,^32^ were found and none of the cats developed FIP, this continuous selection pressure on the virus may successively induce FIPV-specific mutations/glycosylation and/or result in mutations/deletions in no longer required (parts of) proteins, explaining the myriad of genetic changes found in FIP affected cats.

FIP development is characterized by extensive changes in blood leukocytes^13^,^17^,^22^, and the severity of the lymphopaenia strongly determines the outcome of the disease^13^,^17^. In contrast to FIPV, FECV did not induce major changes in peripheral leukocyte subsets, except for a granulocytopaenia in cat 1 during the first 3 weeks after inoculation, and a slight T and B cell lymphocytosis at 56 dpi for cat 1 and 3. This lymphocytosis coincided with the cessation of shedding in these cats. This, together with the fact that shedding did not stop when neutralising antibodies appeared, indicates that the cell-mediated immunity is not only important to overcome FIPV infections, but also to control FECV replication in the gut. FECV infection was characterised by a transient NK cell reduction in peripheral blood, which was most probably the result of migration of NK cells to the intestine or associated lymphoid tissue, since NK cells had an elevated CD11b and CD62L expression (data not shown). FECV infection appeared to be characterised by a transient lowered amount of peripheral Tregs, which can most probably also be explained by specific trafficking to the gut or associated lymphoid tissue. Acute or chronic virus infections are very often associated with an increase in peripheral Treg frequency or function, a feature that was not noticed in the present study. However, gut immunology seems to be substantially different from systemic immunity, notably given the fact that the gut has regulatory systems in place to induce tolerance against commensal bacteria and food antigens, in which Tregs play a vital role. Manipulation of Tregs through accumulation or activation at sites of infection can also cause immune tolerance against pathogenic microorganisms, as exemplified by protozoan (*Leishmania major*), nematodic (*Heligmosomoides polygyrus*) and bacterial (*Helicobacter pylori)* infections^40^,^41^. Whether these cells also contribute to the long-lasting or persistent shedding of FECV remains to be investigated. In addition, cat 2 showed a deviating pattern in peripheral circulating CD8^+^ Tregs compared to the other cats. This subset has gained substantial interest in the context of gut immunity to colorectal cancer, graft-to-host disease and rectal HIV/SIV infection, where they are associated with suppressed immunity^42^,^43^,^44^. However, if and how these cells played a role in the aberrant infection pattern of this cat remains elusive, as not much is known about the exact function of these cells.

In conclusion, the simultaneous assessment of various clinical, virological, and immunological parameters during experimental FECV infection revealed an aberrant infection pattern in one of the cats. Whereas FCoV infections are believed to start with replication in the gut, the aberrant infection pattern shows that FECV has the ability to infect (most probably monocytic) immune cells even in the absence of intestinal replication. In addition, it can be hypothesised that uptake of FECV by mucosa-associated immune cells can induce pressure on the virus to adapt to the replication in these cells, thereby changing some virus’ characteristics, which might give an explanation for the shedding of mutant viruses that completely lacked *in vitro* enterocyte tropism. Based on all these results, it seems that especially acutely infected animals are the major transmitters of FECV. However, given that FIPV arises by mutations and loses its enterocyte tropism, it warrants future research if variant viruses as detected in the present study also occur during natural infections and can increase the odds for FIP to occur, not only within the cat, but potentially also after transmission to other cats, the latter which might give an explanation for infrequently observed epizootics of FIP.

## Methods

### Ethical statement

All experimental procedures were approved by the Local Ethical and Welfare Committee of the Faculty of Veterinary Medicine, Ghent University (EC2012/042), and all methods were carried out in accordance with the approved guidelines.

### Virus

A faecal suspension containing an unknown titre of the FECV strain UCD (originally isolated at UC Davis^1^) was kindly provided by Dr. P. Rottier (Utrecht University, The Netherlands). This suspension was diluted 1/10 in phosphate buffered saline and stored at −70 °C until use. The RNA copy number was determined using an RT-qPCR based on SYBR Green detection, using primers described by Gut *et al.* (1999) (see below). The suspension was centrifuged at 16200 × *g* for 10 min to remove bacterial or host cells, and animals were infected with the suspension supernatant.

### Inoculation and monitoring

Three 14 to 18 months old SPF cats were orally infected with 800 μl of faecal suspension supernatant, containing 10^11.3^ viral RNA copies, while stimulating the swallowing reflex. Cats were housed in the same room but were separated from each other to avoid any physical contact between the animals. Additionally, precautions were taken to prevent exposure to any source of contaminating coronavirus. Briefly, with each handling, sterile clothing and footwear was ensured while litter trays, food trays and water bowls were cleaned and decontaminated daily. To ensure that no contamination could arise from the litter being used, fine sand was washed extensively and autoclaved to serve as litter. The cats were monitored each day during the first week after infection and subsequently on day 9, 14, 21, 28, 56, and 84. Each time, the rectal temperature was measured, lymph nodes were palpated, an oral swab was taken and faeces were collected. If faeces were not available, faecal shedding was monitored by inserting a cotton tipped swab (Copan diagnostics, CA, USA) into the rectum. Swabs were suspended in 1 ml DMEM supplemented with 1000 U ml^−1^ penicillin, 0.4 mg ml^−1^ gentamycin, and 10% fetal bovine serum (FBS). Faeces were diluted 1:5 (w:v) in the same medium. Suspensions were centrifuged (2000 × *g*, 10 min) and supernatant was frozen (−70 °C) until determination of the viral load. Additionally, on day 0, 3, 5, 7, 9, 14, 21, 28, 56 and 84, cats were weighed, and 5 ml blood was taken from the *vena jugularis* in heparin (15 U ml^−1^).

### One step RT-qPCR for the quantification of the viral RNA load

#### RT-qPCR for the detection of total viral RNA (3′ RT-qPCR)

RNA was extracted from the faecal suspension or oral/faecal swab medium using the QIAamp Viral RNA Mini Kit. A one step real-time RT-PCR based on SYBR Green detection was performed with primers described by Gut et al. (1999), targeting a 102 bp fragment at the 3′ end of the genome^23^. A 15 μl PCR mixture was used per reaction and contained 0.3 μl Superscript™ III RT/ Platinum^®^ Taq Mix, 7.5 μl 2x SYBR^®^ Green Reaction Mix with ROX (Superscript™ III Platinum^®^ SYBR^®^ Green One-Step qRT-PCR Kit with ROX, Invitrogen), 0.5 μM forward primer FCoV1128f, 0.5 μM reverse primer FCoV1229r and 3 μl FECV UCD RNA or diluted standard RNA. A reverse transcription step of 20 min at 50 °C and a denaturation step at 95 °C for 5 min were followed by 45 cycles each 15 s at 95 °C and 30 s at 60 °C. A first-derivative melting curve analysis was performed by heating the mixture to 95 °C for 15 s and then cooling to 60 °C for 1 min and heating back to 95 °C at 0.3 °C increments. Reverse transcription, amplification, monitoring and melting curve analysis were carried out in a Step One Plus™ real-time PCR system. Ten-fold serial dilutions of cRNA standards were made over a dynamic range of 6 log units (10^7^–10^2^) for the generation of the standard curve, showing an efficiency of 92.41 ± 1.02%, R^2^ values of 0.99, and Y-intercept values of 44.87 ± 1.11.

#### RT-qPCR for the detection of genomic RNA (5′ RT-qPCR)

RNA was extracted from the faecal suspensions using the QIAamp Viral RNA Mini Kit. Primer design and PCR conditions have previously been described^21^. Ten-fold serial dilutions of cRNA standards were made over a dynamic range of 6 log units (10^7^–10^2^) for the generation of the standard curve, showing an efficiency of 93.96 ± 0.76%, R^2^ values of 0.99, and Y-intercept values of 37.61 ± 0.93.

### Infectivity titration

Infectivity titrations were performed in feline colonocytes, as previously described^21^.

### Determination of neutralising serum antibody titres

Sera were incubated at 56 °C for 30 min to inactivate complement. Two-fold serial dilutions of the sera were mixed with an equal volume of a virus suspension containing 100 TCID_50_ FECV UCD and incubated for 1 h (37 °C, 5% CO_2_). Then, colonocytes were added and further incubated with the virus-serum suspensions for 3 days. Infection was visualised by means of immunoperoxidase monolayer assay, as previously described^21^. The virus neutralising titres were expressed as the reciprocal of the serum dilution that neutralised infection in 50% of the monolayers.

### Leukocyte isolation

Blood mononuclear cells were separated on Ficoll-Paque. Maximum 2 × 10^7^ cells ml^−1^ were resuspended in RPMI supplemented with 30% FBS, 100 U penicillin ml^−1^, 0.1 mg streptomycin ml^−1^, and 10% dimethyl sulfoxide (DMSO). Subsequently, cells were frozen by lowering the temperature with 1 °C min^−1^ until −30 °C, followed by a 15 min incubation period at −30 °C and finally lowering the temperature to −150 °C at a rate of 1 °C s^−1^. Next, cells were stored in liquid nitrogen.

### Antibodies used for leukocyte staining

Monoclonal antibodies against the epsilon chain of feline CD3 (NZM1) and against feline CD56 (SZK1) were kindly provided by Dr. Yorihiro Nishimura (Tokyo University, Japan)^45^. Monoclonal antibodies FE5.4D2, and CA2.1D6 recognising feline CD8β, and canine CD21, respectively, were purchased from Bio-Rad. A monoclonal antibody (FJK-16s), directly conjugated with Alexa fluor 647 (AF647) and cross-reacting with feline Foxp3 was purchased from eBioscience. Monoclonal antibody CAT30A against feline CD4 was purchased from Veterinary Medical Research and Development (VMRD). Conjugated secondary antibodies (Invitrogen) were goat anti-rat Alexa Fluor 488, goat anti-mouse IgG R-Phycoerythrin, goat anti-mouse IgG2a Alexa Fluor 488, goat anti-mouse IgG1 Alexa Fluor 647 and goat anti-mouse IgG3 fluorescein isothiocyanate (FITC). When primary antibodies from the same IgG1 isotype were used, one primary antibody was labeled with Zenon Alexa Fluor 488 Mouse IgG1.

### Leukocyte staining

Phenotyping of cells was performed simultaneously. All analysed cells were first stored in liquid nitrogen, facilitating analysis workflow. Several precautions were taken in order to preserve immunophenotypic properties as was done in previous research^46^. Briefly, cells were frozen directly after isolation, they were stored at −196 °C for the entire storage period and viability of thawed cells was 80–90%. A minimum of 1 × 10^6^ of frozen cells were stained for phenotypic analysis in RPMI supplemented with 1 mM Ethylenediaminetetraacetic acid (EDTA). Cells were incubated for 20 min at 4 °C while gently shaking, both with the primary and dye-conjugated secondary antibodies. Cells were washed with cold RPMI containing EDTA and centrifuged at 300 × *g* for 10 min at 4 °C. During regulatory T cell staining, surface molecules were first stained, after which cells were fixed with the fixation/permeabilisation kit optimised for staining of intracellular Foxp3 (eBioscience). Cells were then stained with anti-Foxp3 antibody, directly conjugated with AF647. Analysis was done on a FACSCanto flow cytometer using FACSDiva software. After singlet gating, a minimum of 2 × 10^5^ events was analysed.

### Illumina sequencing of faecal samples

Faecal suspensions were filtered twice using 0.8 μm and 0.45 μm membrane filters. Two microliter of Benzonase Nuclease (Novagen), 1 μl of Micrococcal Nuclease (New England Biolabs) and 1 μl of NEBNext® RNase III RNA Fragmentation Module (New England Biolabs) in 7 μl of homemade buffer (1 M Tris, 100 mM CaCl_2_ and 30 mM MgCl_2_, pH = 8) were added to 140 μl of faecal filtrate, and incubated for 2 h at 37 °C to destroy free and bacterial DNA/RNA. Next, seven microliter of 0.2 M EDTA was added to the sample for enzyme inactivation. Extraction of viral RNA was performed using the QIAamp Viral RNA Mini Kit according to the manufacturer’s instructions, but without adding carrier RNA. Total RNA was converted into cDNA using the Whole Transcriptome Amplification Kit (Sigma Aldrich). Therefore, 0.5 μl Library Synthesis Solution was added to 2.82 μl of RNA, followed by denaturation for 2 min at 95 °C. RNA was cooled to 18 °C and 0.5 μl Library Synthesis Buffer, 0.4 μl Library Synthesis Enzyme and 0.78 μl of water was immediately added to the reaction. The mixture was subjected to the following temperature profile: 18 °C, 25 °C, 37 °C, 42 °C, and 70 °C for 10, 10, 30, 10, and 20 minutes, respectively. Samples were cooled down to 4 °C followed by a brief centrifugation step. A mastermix containing 60.2 μl of nuclease free water, 7.5 μl of Amplification Mix, 1.5 μl of WTA dNTP mix and 0.75 μl Amplification Enzyme was added to the sample and incubated as follows: 94 °C for 2 min and 30 cycles at 94 °C for 30 s and 70 °C for 5 min. WTA products were purified with the MSB^®^ Spin PCRapace kit (Stratec) according to the manufacturer’s instructions and prepared for Illumina sequencing using the KAPA Library Preparation Kit (Kapa Biosystems), according to the instructions of the manufacturer.

Fragments ranging from 350–600 bp were selected using the BluePippin (Sage Science) according to the manufacturer’s instructions. Sequencing of the samples was performed on a HiSeq™ 2500 platform (Illumina) for 300 cycles (150 bp paired ends). Raw reads were trimmed for quality and adapters, and were *de novo* assembled into contigs using SPAdes^47^. Scaffolds were classified using a tBLASTx search against all complete viral genomes in GenBank using an e-value cut-off of 10^−10^. Scaffolds with a significant tBLASTx hit were retained and used for a second tBlastx search against the GenBank nucleotide database using an e-value of 10^−4^. The obtained consensus sequences, and the identified deletions in the complete FECV genomes were checked and curated by mapping the trimmed reads back to the obtained consensus sequences using BWA^48^.

## Additional Information

**How to cite this article**: Desmarets, L. M.B. *et al.* Experimental feline enteric coronavirus infection reveals an aberrant infection pattern and shedding of mutants with impaired infectivity in enterocyte cultures. *Sci. Rep.* **6**, 20022; doi: 10.1038/srep20022 (2016).

## Figures and Tables

**Table 1:** Detection of viraemia during the entire infection course.

Day pi	Cat 1		Cat 2		Cat 3	
	Plasma	Cell- associated	Plasma	Cell- associated	Plasma	Cell- associated
0	—	—	—	—	—	—
3	—	—	—	Cq 34.2	—	—
5	—	—	—	Cq 32.7	—	Cq 36.1
7	—	Cq 35.8	—	—	—	—
9	—	Cq 29.3	—	—	—	Cq 37
14	—	Cq 36.5	—	—	—	Cq 32.4
21	—	—	—	—	—	—
28	—	—	—	—	—	Cq 37.6
56	—	—	—	—	—	—
84	—	—	—	—	—	—

**Table 2:** Nucleotide and amino acid changes in the viral genome during early shedding.

Sample	Affected nt position (compared to the inoculum^1^)	Protein	Type of nt change	Amino-acid change
Cat 1 day 7^2^	14284	nsp12	A > G	no
	15049	nsp12	A > G	no
Cat 2 day 21^3^	428	nsp1	C > T	no
	763	nsp2	A > G	K159R^5^
	9100	nsp5	C > T	S2938L^6^
	9880	nsp6	C > T	S3198F^7^
	17329	nsp14	T > C	no
	28796–28896	7b	101bp deletion	Y142L, H143L, early stop (143 aa)^8^
Cat 3 day 9^4^	6287	nsp3	C > T	no
	9880	nsp6	C > T	S3198F^9^
	11647	nsp9	A > G	D3787G^10^
	12844	nsp12	T > C	no
	12846	nsp12	T > C	I171T^11^
	21925	Spike S1	G > T	E518Y^12^
	21927	Spike S1	G > T	E518D^13^

^1^GenBank accession number KU215419.

^2^GenBank accession number KU215420.

^3^GenBank accession numbers KU215421 (with 7b deletion) and KU215422 (without 7b deletion).

^4^GenBank accession number KU215423.

^5^82.4%, ^6^62.3%, ^7^44.6%, ^8^83.8%, ^9^31%, ^10^21.9%, ^11^40%, ^12,13^32.7% of all reads.

**Table 3:** Nucleotide and amino acid changes in the viral genome at day 28 (cat 1 and 3) or 84 pi (cat 2).

Sample	Affected nt position (compared to the inoculum^1^)	Protein	Type of nt change	Amino acid change
Cat 1 day 28^2^	2986	nsp3	G > A	R900H^5^
	14284	nsp12	A > G	no
	15049	nsp12	A > G	no
	20470	Spike S1	C > T	P33S^6^
	20609	Spike S1	A > C	H79P^7^
	20932..20940	Spike S1	9bp deletion	del aa186-188^8^
	22127	Spike S1	A > G	H585R^9^
	22368	Spike S1	G > T	K665N^10^
	27429	Nucleocapsid	T > G	V169G^11^
	28770	7b	C > A	no
	28797	7b	C > T	no
	28803	7b	C > T	no
Cat 2 day 84^3^	763	nsp2	A > G	K159R^12^
	1946	nsp2	C > T	no
	9880	nsp6	C > T	S3198F^13^
	13018	nsp12	C > T	no
	18829	nsp15	A > G	no
	20471	Spike S1	C > T	P33L^14^
	20570	Spike S1	G > A	G66D^15^
	20615	Spike S1	G > A	G81E^16^
	20695	Spike S1	G > A	E108K^17^
	20726	Spike S1	G > A	R118H^18^
	20846	Spike S1	A > G	K158R^19^
	20872	Spike S1	C > T	R/Q167W^20^
	22368	Spike S1	G > T	K665N^21^
	23693	Spike S2	C > T	T1107I^22^
	26231	Membrane	G > A	E37K^23^
	28785	7b	A > G	no
Cat 3 day 28^4^	428	nsp1	C > T	no
	3060	nsp3	A > G	K925E^24^
	6614	nsp3	T > C	no
	9880	nsp6	C > T	S3198F^25^
	17329	nsp 14	T > C	no
	20465..20488	Spike S1	24 bp deletion	del aa31-38; F39I^26^
	20470	Spike S1	C > T	P33S^27^
	20702	Spike S1	A > C/T	D110A/D110V^28^
	21076	Spike S1	C > A	depends on nt 21077
	21077	Spike S1	A > G	Q235K/G^29^
	21558	Spike S1	C > T	no
	22084	Spike S1	C > T	P571S^30^
	23135	Spike S2	A > C	N921T^31^
	27199	Nucleocapsid	T > C	no
	28805	7b	A > G	Q145R^32^

^1^GenBank accession number KU215419.

^2^GenBank accession numbers KU215424 (with S1 deletion) and KU215425 (without S1 deletion).

^3^GenBank accession number KU215426.

^4^GenBank accession numbers KU215427 (with S1 deletion) and KU215428 (without S1 deletion).

^5,6,7,9,12,13,14,15,16,17,18,20,21,22,23,28^100%, ^8^97.8%, ^10^12.1%, ^11^18.6%, ^19^79.3%, ^24^24.3%, ^25^36.8%, ^26^90%, ^27^10%, ^29^38.8%, 3015.5%, 3113.9%, 3257.9% of all reads.
